# Phosphonamidates are the first phosphorus-based zinc binding motif to show inhibition of β-class carbonic anhydrases from bacteria, fungi, and protozoa

**DOI:** 10.1080/14756366.2019.1681987

**Published:** 2019-10-30

**Authors:** Siham A. Alissa, Hanan A. Alghulikah, Zeid A. Alothman, Sameh M. Osman, Sonia Del Prete, Clemente Capasso, Alessio Nocentini, Claudiu T. Supuran

**Affiliations:** a Chemistry Department, College of Science, Princess Nourah bint Abdulrahman University, Riyadh, Saudi Arabia; b Chemistry Department, College of Science, King Saud University, Riyadh, Saudi Arabia; c Istituto di Bioscienze e Biorisorse, CNR, Napoli, Italy; d NEUROFARBA Department, Sezione di Scienze Farmaceutiche e Nutraceutiche, Sezione di Scienze Farmaceutiche e Nutraceutiche, Università degli Studi di Firenze, Sesto Fiorentino (Firenze), Italy

**Keywords:** Virulence, resistance, carbonic anhydrase, inhibition, phosphonamidate, selectivity

## Abstract

A primary strategy to combat antimicrobial resistance is the identification of novel therapeutic targets and anti-infectives with alternative mechanisms of action. The inhibition of the metalloenzymes carbonic anhydrases (CAs, EC 4.2.1.1) from pathogens (bacteria, fungi, and protozoa) was shown to produce an impairment of the microorganism growth and virulence. As phosphonamidates have been recently validated as human α-CA inhibitors (CAIs) and no phosphorus-based zinc-binding group have been assessed to date against β-class CAs, herein we report an inhibition study with this class of compounds against β-CAs from pathogenic bacteria, fungi, and protozoa. Our data suggest that phosphonamidates are among the CAIs with the best selectivity for β-class over human isozymes, making them interesting leads for the development of new anti-infectives.

## 1. Introduction

Virulence is labelled as the ability of a microorganism to infect the host and cause a disease. Virulence factors are molecules that assist the pathogen to colonise the host at the cellular level^1^. These factors can be either secretory, membrane associated or cytosolic and facilitate the microorganism metabolic, physiological, and morphological adaption in the host^1^^,^^2^.

Virulence factors are deeply implicated in the onset of pharmacological resistance to anti-infectives. Preventing the implementation of these processes is a strategy deemed successful to date to overcome drug-resistance^3^. Targeting virulence factors might indeed reduce, eliminate, and/or reverse the evolutionary selective pressures that induce the pathogen to develop resistance, which represents a main threat to human health nowadays. The metalloenzymes carbonic anhydrases (CAs, EC 4.2.1.1) have been shown to play critical roles in the virulence of many pathogens among which bacteria, fungi, and protozoa^4^^,^^5^.

CAs are a superfamily of phylogenetically ubiquitous metalloenzymes, present in Prokaryotes and Eukaryotes, classified in seven genetically unrelated families, α, β, γ, δ, ζ, η, and θ^4–8^. By catalysing the reversible hydration of CO_2_ to HCO_3_^−^ and H^+^, these enzymes are implicated in many physiological processes which are basic for life^5^^,^^9^. In microorganisms, CAs are involved in photosynthesis (cyanobacteria), biosynthesis of DNA, amino acids and fatty acids, and proliferation, survival and differentiation of pathogens both in the hosts and in the environmental niches^4^^,^^10^. Compelling data exist in the literature, which strongly indicate that interference with CA activity in various parasites leads to an impairment of their growth, which in turn leads to a significant anti-infective effect^10^.

Unlike mammals and human whose genome uniquely encode for α-class CAs, the genome of many pathogenic bacteria (e.g. *Vibrio cholerae*^11^, *Francisella tularensis*^12^, *Burkholderia pseudomalei*^13^, the Gram-negative bacteria provoking cholera, tularaemia, and melioidosis), fungi (e.g. *Candida glabrata*^14^, *Cryptococcus neoformans*^15^, or *Malassezia globosa*^16^, respectively responsible of candidiasis, cryptococcosis, and dandruff), and protozoa (e.g. *Leismania donovani chagasi* which provokes leishmaniasis^17^) encode for β-class enzymes. Actually, the seven different CA classes do not show significant structural homology with each other. Furthermore, CAs of the same class but belonging to genetically distant species, show differences at the level of their catalytic site^18^. Therefore, a probable and realistic possibility to develop selective CA inhibitors exists. β-CAs exist as oligomers formed by two or more identical subunits such as dimers, tetramers, and octamers^19^.

Sulphonamides and bioisosteres are the most potent class of CA inhibitors (CAIs) and have been, probably exhaustively, exploited to date in the design of inhibitors against almost every known CA^9^. Nonetheless, these chemotypes commonly show a weak isoform-selectivity both within the subset of human CAs and between isoforms from distinct classes, such as α- and β-^5^. As a result, many other chemotypes (phenols^20^^,^^21^, mono- and dithiocarbammates^22^^,^^23^, boroles^24^, carboxylates,^25^ N-nitrosulfonamides^26^^,^^27^) have been investigated as inhibitors of β-CAs to identify selective modulators for targeting the pathogens encoding for enzymes of this class. Among these, N-nitrosulfonamides, phenols, and natural polyphenols stood out as selective inhibitors for β-class CAs from pathogens over human ubiquitous CAs^20^^,^^21^^,^^27^.

**Figure 1.**
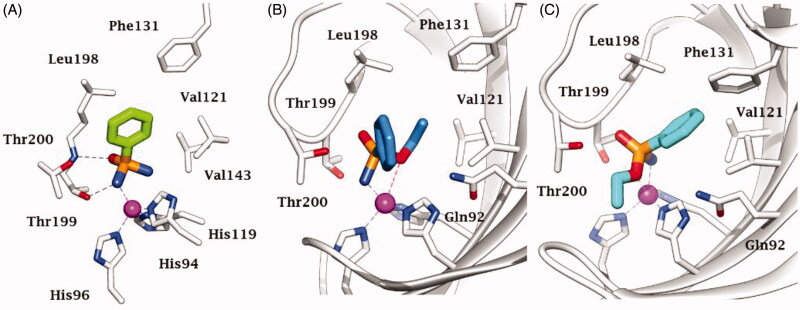
*In silico* predicted binding mode of (A) phenylphosphonic diamide **2**, (B) *(S)*-**4** and (C) *(R)*-**4** to hCA II, as repoted in Nocentini et al.^28^

A recent paper by us showed for the first time that phosphonamidates are able to inhibit human CAs in the micromolar range acting a sulphonamide biomimetics additionally possessing a chiral binding mode (Figure 1)^28^. Up to now, no one scheduled testing phosphorus based binding motifs against β-class CAs. Considering the interesting results reported earlier against these enzymes, here we report an inhibition study with benzenephosphonamidates against β-CAs from pathogenic bacteria, fungi, and protozoa working out their structure–activity relationship (SAR) in comparison to hCA I and II previously reported inhibition.

## 2. Methods

### 2.1. Chemistry

The synthesis of phenylphosphonic diamide **2** and alkyl phosphonamidates **3**–**10** was reported earlier by our group^28^.

### 2.2. Carbonic anhydrase inhibition

An Sx.18Mv-R Applied Photophysics (Oxford, UK) stopped-flow instrument has been used to assay the catalytic activity of various CA isozymes for CO_2_ hydration reaction^29^. Phenol red (at a concentration of 0.2 mM) was used as indicator, working at the absorbance maximum of 557 nm, with 10 mM Hepes (pH 7.5, for α-CAs) or TRIS (pH 8.3, for β-CAs) as buffers, and 20 mM Na_2_SO_4_ (for maintaining constant the ionic strength), following the initial rates of the CA-catalysed CO_2_ hydration reaction for a period of 10–100 s. The CO_2_ concentrations ranged from 1.7 to 17 mM for the determination of the kinetic parameters and inhibition constants. For each inhibitor, at least six traces of the initial 5–10% of the reaction have been used for determining the initial velocity. The uncatalyzed rates were determined in the same manner and subtracted from the total observed rates. Stock solutions of inhibitor (0.1 mM) were prepared in distilled–deionised water and dilutions up to 0.01 nM were done thereafter with the assay buffer. Inhibitor and enzyme solutions were preincubated together for 1 h at room temperature prior to assay, in order to allow for the formation of the E–I complex. The inhibition constants were obtained by nonlinear least-squares methods using PRISM 3 and the Cheng–Prusoff equation, as reported earlier, and represent the mean from at least three different determinations^30^^,^^31^. All CA isoforms were recombinant ones obtained in-house as reported earlier^32^^,^^33^.

## 3. Results and discussion

### 3.1. Chemistry

**Scheme 1.**
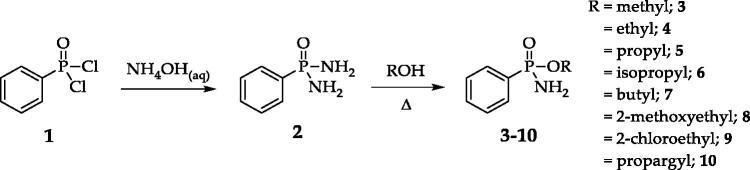
Synthesis of benzenephosphonamidate derivatives.

A straightforward strategy was used to synthesise the compounds starting from phenylphosphonic dichloride **1**^28^ (Scheme 1). Phenylphosphonic diamide **2** was obtained by reacting 1 with an aqueous solution of 35% ammonia. Derivative **2** was converted to alkyl phosphonamidates **3–10** by reaction with the proper alcohol, as described in the literature^34^ (Scheme 1). Unlike the diamide derivative **2**, all alkyl phosphonamidates **3–10** were obtained as racemic mixtures of R and S optical isomers.

### 3.2. β-Class carbonic anhydrase inhibition

Phenylphosphonic diamide **2** and alkyl phosphonamidates **3–10** were assayed as inhibitors of a panel of β-class CAs from pathogenic bacteria, fungi, and protozoa: VChβ from *V. cholerae*, FtuβCA from *F. tularensis*, BpsCAβ from *B. pseudomalei*, Can2 from *C. neoformans*, CgNce from *C. glabrata*, MgCA from *M. globosa*, and LdcCA from *L. donovani chagasi.* A stopped flow CO_2_ hydrase assay was used including acetazolamide (**AAZ**) as standard inhibitor^29^. A longer preincubation between compounds **2–10** and the enzymes (1 h) was necessary to observe their maximum inhibitory activity in comparison to the 15 min incubation which is commonly used for sulphonamides inhibitors. It was previously shown that phosphonamidates possess a significantly lower acidity (more than 2 orders of magnitude) than the bioisosteres sulphonamides which presumably induce an extended time needed to form the E–I complex.

The inhibition profiles against the human ubiquitous CAs I and II are displayed for comparison. The following structure–activity relationships (SAR) can be assembled from the inhibition data reported in Table 1.

**Table 1.** Inhibition data of VChβ, FtuβCA, BpsCAβ, Can2, CgNce, MgCA and LdcCA with phenylphosphonic diamide **2** and alkyl phosphonamidates **3–10** and the standard sulphonamide inhibitor acetazolamide (**AAZ**) by a stopped flow CO_2_ hydrase assay^29^.

		K_I_ (µM)^a^								
Compound	R	VChβ	FtuβCA	BpsCAβ	Can2	CgNce	MgCA	LdcCA	CA I	CA II
**2**	–	0.9	10.3	2.5	0.03	0.05	28.4	2.1	77.8	32.8
**3**	–CH_3_	0.5	8.5	2.1	0.02	0.2	26.1	0.8	145.6	39.8
**4**	–CH_2_CH_3_	9.5	16.4	4.6	0.05	0.8	68.1	3.5	338.6	160.6
**5**	–(CH_2_)_2_CH_3_	15.4	35.6	12.6	0.9	1.3	79.0	6.8	589.9	459.4
**6**	–CH(CH_3_)_2_	7.3	18.4	5.8	0.1	0.7	52.4	5.1	730	348.8
**7**	–(CH_2_)_3_CH_3_	26.8	42.0	22.5	2.2	5.3	102.4	16.4	876.3	750
**8**	–(CH_2_)_2_OCH_3_	64.9	55.3	29.4	10.4	8.7	265.7	36.5	961.2	520.1
**9**	–(CH_2_)_2_Cl	34.7	15.1	32.7	0.6	1.8	117.3	26.4	322.2	95.4
**10**	–CH_2_CCH	20.9	6.9	18.9	5.3	20.2	97.4	19.3	575.8	465.3
**AAZ**	–	0.45	0.77	0.74	0.01	0.01	40	0.09	0.25	0.01

^a^ Mean from three different assays, by a stopped flow technique (errors were in the range of ±5–10% of the reported values).

What immediately stands out from the data in Table 1 is that benzenephosphonamidates **2–10** act as significantly stronger inhibitors against the tested β-class CAs than CA I and CA II. In fact, low to submicromolar inhibition constants (K_I_s) were measured against VChβ (K_I_s = 0.5–64.9 µM), FtuβCA (K_I_s = 6.9–55.3 µM), BpsCAβ (K_I_s = 2.1–32.7 µM), Can2 (K_I_s = 0.02–10.4 µM), CgNce (K_I_s = 0.05–20.2 µM), and LdcCA (K_I_s = 0.8–36.5 µM), whereas solely MgCA was inhibited in a medium micromolar range (K_I_s = 26.1–265.7 µM). In contrast, CAs I and II had been shown to be target of compounds **2–10** in a medium to high micromolar scale spanning from 77.8 to 961.2 µM and 32.8 to 520.1 µM, respectively.

Using *in silico* techniques, phosphonamidates have been reported to act as zinc-binding group in the interaction with the active site of hCA II (Figure 1) and thus it is not unexpected that the incorporation of gradually bulkier substituents on the phosphorus atom might decrease the inhibitory effectiveness of this class of compounds because of steric hindrance induced nearby the zinc ion. Nonetheless, the reduction of inhibition potency against β-class CAs upon increasing the length of the alkyl chain was not as significant as that reported against hCAs, and thus other binding factors appear to take place within the binding site of β-CAs. In detail, phenylphosphonic diamide **2** and methyl phosphonamidate **3** indistinctly showed the best inhibitory potency against VChβ (K_I_s of 0.9 and 0.5 µM), BpsCAβ (K_I_s of 2.5 and 2.1 µM), Can2 (K_I_s of 0.03 and 0.02 µM), CgNce (K_I_s of 0.05 and 0.2 µM), MgCA (K_I_s of 28.4 and 26.1 µM), and LdcCA (K_I_s of 2.1 and 0.8 µM) among the reported compounds. As a unique exception, FtuβCA was found to be most potently inhibited by the propargyl phosphonamidate **10** which shows a K_I_ of 6.9 µM. In the cases of BpsCAβ and Can2, the ethyl derivative **4** showed a comparable inhibition to compounds **2** and **3**, with K_I_s of 4.6 and 0.05 µM, and further showed an interesting submicromolar K_I_ of 0.8 µM against CgNce. Branching of the alkyl chain from propyl (**5**) to isopropyl (**6**) always produced a positive effect on the inhibition potency, which was two-fold against VChβ, FtuβCA, BpsCAβ, and CgNce and nine-fold against Can2 (K_I_ from 0.9 to 0.1 µM). Switching the propyl (**6**) to a propargyl (**10**) produced instead a positive effect uniquely in the case of FtuβCA (K_I_ from 35.6 to 6.9 µM). Enhancement of inhibitory potency against FtuβCA (K_I_ from 35.6 to 15.1 µM) and Can2 (K_I_ from 0.9 to 0.6 µM) is also provoked by swapping the terminal CH_3_ of the propyl of **5** with a chlorine atom as in **9**, whereas the two compounds inhibit CgNce almost equally (K_I_s of 1.3 and 1.8 µM).

The standard **AAZ** is a more effective inhibitor of all tested β-CAs than phosphonamidates **2–10** here investigated (K_I_s in the range 0.01–0.77 µM), with the exception of MgCA, against which it showed a K_I_ of 40 µM, whereas the most potent compounds **2** and **3** act with K_I_s of 28.4 and 26.1 µM. However, **AAZ** also inhibits CA I and II in a medium to low nanomolar range, while K_I_s of compounds **2–10** do not go below 30 µM against these human isozymes. In Table 2, the selectivity index (SI) for the target β-CAs over hCA II is reported. With the exception of MgCA, all tested phosphonamidates show a remarkably selective inhibitory action against all β-CAs over the most relevant human isozyme. While the SI for VChβ, FtuβCA, LdcCA and BpsCAβ (over hCA II) span between 2.7 and 79.6, it is noteworthy stressing the up to four-digits SI calculated for a subset of compounds against Can2 (1093.3–3488.0) and the three-digits SI that compounds **2–7** showed against CgNce (141.5–656.0).

**Table 2.** Selectivity index (SI) for target β-CAs over hCA II.

		SI (K_I_ CA II/K_I_ β-CA)						
Compound	R	VChβ	FtuβCA	BpsCAβ	Can2	CgNce	MgCA	LdcCA
**2**	–	36.4	3.2	13.1	1093.3	656.0	1.2	15.6
**3**	–CH_3_	79.6	4.7	19.0	1990.0	199.0	1.5	49.8
**4**	–CH_2_CH_3_	16.9	9.8	34.9	3212.0	200.8	2.4	45.9
**5**	–(CH_2_)_2_CH_3_	29.8	12.9	36.5	510.4	353.4	5.8	67.6
**6**	–CH(CH_3_)_2_	47.8	19.0	60.1	3488.0	498.3	6.7	68.4
**7**	–(CH_2_)_3_CH_3_	28.0	17.9	33.3	340.9	141.5	7.3	45.7
**8**	–(CH_2_)_2_OCH_3_	8.0	9.4	17.7	50.0	59.8	2.0	14.2
**9**	–(CH_2_)_2_Cl	2.7	6.3	2.9	159.0	53.0	0.8	3.6
**10**	–CH_2_CCH	22.3	67.4	24.6	87.8	23.0	4.8	24.1
**AAZ**	–	0.02	0.01	0.01	1.0	1.0	/	/

**AAZ** weakly inhibits MgCA from *M. globosa* whereas phosphonamidates **2–10** inhibit this enzyme less effectively than other screened β-CAs and almost comparably with hCA II, leading to SI ranging between 0.8 and 7.3. Selectivity indexes for the target β-CA over hCA I are obviously even higher than those depicted in Table 2 because hCA I is less inhibited than hCA II by derivatives **2–10**.

Significantly higher SI have been calculated with most phosphonamidates for β-class CAs over human isoform II in comparison to sulphonamide derivatives^35^, which are here represented by **AAZ** (SI in the range <0.01–1.0, Table 2). These data include this new class of compounds among the CAIs most selective for β-class over human isozymes known to date. One could speculate that the narrower active site pocket of β-CAs better accommodates benzenephosphonamidates than the roomier binding cavity of human CAs in terms of binding interactions. Indeed, a recent paper by us showed that the binding mode of benzoxaboroles within the active site of β-CAs Can2 and MgCA is strongly driven by π–π interactions involving aromatic residues solely present in β-class enzymes^19^. Analogue binding interactions might exist with benzenephosphonamidates, which increase their inhibition effectiveness against β-CAs. Novel insights are emerging by novel crystallographic studies currently ongoing with phosphonamidates and hCAs, which will definitively clarify the binding mode of such a chemotype with these metalloenzymes. Therefore, this knowledge will be extended to visualise the reasons for benzenephosphonamidates selective action against β- over α-class enzymes.

## 4. Conclusions

The robust spread of antimicrobial resistance represents a main threat to human health. A primary strategy to combat it consists in the identification of novel therapeutic targets and anti-infectives with alternative mechanisms of action. The inhibition of the metalloenzymes carbonic anhydrases (CAs, EC 4.2.1.1) from pathogens among bacteria, fungi, and protozoa was shown to produce an impairment of the microorganism growth and virulence, whose targeting is deemed to prevent the evolutionary selective pressures that induce the pathogen to develop resistance. Unlike mammals and thus human, the genome of many pathogenic bacteria, fungi, and protozoa encode for β-class enzymes, which show significant structural diversities compared to α-class isozymes. As phosphonamidates have been recently validated as human α-CA inhibitors (CAIs) and no phosphorus-based zinc-binding group have been assessed to date against β-class CAs, herein we report an inhibition study with this class of compounds against β-CAs from pathogenic bacteria, fungi, and protozoa. Compounds **2–10** showed low to submicromolar K_I_s against most tested β-CAs, namely VChβ (K_I_s = 0.5–64.9 µM), FtuβCA (K_I_s = 6.9–55.3 µM), BpsCAβ (K_I_s = 2.1–32.7 µM), Can2 (K_I_s = 0.02–10.4 µM), CgNce (K_I_s = 0.05–20.2 µM), and LdcCA (K_I_s = 0.8–36.5 µM). As human isoforms CAs I and II are instead solely inhibited in a high micromolar range (32.8–961.2 µM), these data include phosphonamidates among the CAIs most selective for β-class over human isozymes known to date, making them interesting leads for the development of new anti-infective agents. Crystallographic studies currently ongoing with phosphonamidates **2–10** and hCAs will definitively clarify the mechanism of action of this class of compounds probably shedding light on their selective efficacy against β- over α-class enzymes

## References

[1] Allen RC, Popat R, Diggle SP, et al. Targeting virulence: can we make evolution-proof drugs? Nat Rev Microbiol 2014;12:300–8. 24625893 10.1038/nrmicro3232

[2] Clatworthy AE, Pierson E, Hung DT. Targeting virulence: a new paradigm for antimicrobial therapy. Nat Chem Biol 2007;3:541–8. 17710100 10.1038/nchembio.2007.24

[3] (a) Du Toit A. Targeting virulence. Nat Rev Microbiol 2015;13:2; 10.1038/nrmicro3418 25534810

[4] (a) Capasso C, Supuran CT. Bacterial, fungal and protozoan carbonic anhydrases as drug targets. Expert Opin Ther Targets. 2015;149:1689–1704; 10.1517/14728222.2015.1067685 26235676

[5] (a) Supuran CT. Carbonic anhydrases: novel therapeutic applications for inhibitors and activators. Nat Rev Drug Discovery 2008;7:168–81; 18167490 10.1038/nrd2467

[6] Xu Y, Feng L, Jeffrey PD, et al. Structure and metal exchange in the cadmium carbonic anhydrase of marine diatoms. Nature 2008;452:56–61. 18322527 10.1038/nature06636

[7] Lane TW, Saito MA, George GN, et al. Biochemistry: a cadmium enzyme from a marine diatom. Nature 2005;435:42. 15875011 10.1038/435042a

[8] Del Prete S, Vullo D, Fisher GM, et al. Discovery of a new family of carbonic anhydrases in the malaria pathogen Plasmodium falciparum. The η-carbonic anhydrases. Bioorg Med Chem Lett 2014;18:4389–96. 10.1016/j.bmcl.2014.08.015 25168745

[9] (a) Alterio V, Di Fiore A, D’Ambrosio K, et al. Multiple binding modes of inhibitors to carbonic anhydrases: how to design specific drugs targeting 15 different isoforms?. Chem Rev 2012;112:4421–68; 22607219 10.1021/cr200176r

[10] Supuran CT, Capasso C. Biomedical applications of prokaryotic carbonic anhydrases. Expert Opin Ther Pat 2018;28:745–54. 29973089 10.1080/13543776.2018.1497161

[11] Del Prete S, Vullo D, De Luca V, et al. Sulfonamide inhibition studies of the β-carbonic anhydrase from the pathogenic bacterium *Vibrio cholerae*. Bioorg Med Chem 2016;24:1115–20. 26850377 10.1016/j.bmc.2016.01.037

[12] Del Prete S, Vullo D, Osman SM, et al. Sulfonamide inhibition profiles of the β-carbonic anhydrase from the pathogenic bacterium *Francisella tularensis* responsible of the febrile illness tularemia. Bioorg Med Chem 2017;25:3555–61. 28511911 10.1016/j.bmc.2017.05.007

[13] Vullo D, Del Prete S, Osman SM, et al. Comparison of the amine/amino acid activation profiles of the β- and γ-carbonic anhydrases from the pathogenic bacterium Burkholderia pseudomallei. J Enzyme Inhib Med Chem 2018;33:25–30. 29098887 10.1080/14756366.2017.1387544 PMC6009869

[14] Cottier F, Leewattanapasuk W, Kemp LR, et al. Carbonic anhydrase regulation and CO_2_ sensing in the fungal pathogen Candida glabrata involves a novel Rca1p ortholog. Bioorg Med Chem 2013;21:1549–54. 22727373 10.1016/j.bmc.2012.05.053

[15] Schlicker C, Hall RA, Vullo D, et al. Structure and inhibition of the CO_2_-sensing carbonic anhydrase Can2 from the pathogenic fungus *Cryptococcus neoformans*. J Mol Biol 2009;385:1207–20. 19071134 10.1016/j.jmb.2008.11.037

[16] Hewitson KS, Vullo D, Scozzafava A, et al. Molecular cloning, characterization, and inhibition studies of a beta-carbonic anhydrase from *Malassezia globosa*, a potential antidandruff target. J Med Chem 2012;55:3513–20. 22424239 10.1021/jm300203r

[17] Syrjänen L, Vermelho AB, Rodrigues Ide A, et al. Cloning, characterization, and inhibition studies of a β-carbonic anhydrase from *Leishmania donovani chagasi*, the protozoan parasite responsible for leishmaniasis. J Med Chem 2013;56:7372–81. 23977960 10.1021/jm400939k

[18] Lomelino CL, Andring JT, McKenna R. Crystallography and its impact on carbonic anhydrase research. Int J Med Chem 2018;2018:9419521. 30302289 10.1155/2018/9419521 PMC6158936

[19] Nocentini A, Cadoni R, Del Prete S, et al. Benzoxaboroles as efficient inhibitors of the β-carbonic anhydrases from pathogenic fungi: activity and modeling study. ACS Med Chem Lett 2017;8:1194–8. 29152053 10.1021/acsmedchemlett.7b00369 PMC5682618

[20] Entezari Heravi Y, Bua S, Nocentini A, et al. Inhibition of *Malassezia globosa* carbonic anhydrase with phenols. Bioorg Med Chem 2017;25:2577–82. 28343756 10.1016/j.bmc.2017.03.026

[21] Nocentini A, Bua S, Del Prete S, et al. Natural polyphenols selectively inhibit β-carbonic anhydrase from the dandruff-producing fungus *Malassezia globosa*: activity and modeling studies. ChemMedChem 2018;13:816–23. 29575699 10.1002/cmdc.201800015

[22] Vullo D, Del Prete S, Nocentini A, et al. Dithiocarbamates effectively inhibit the β-carbonic anhydrase from the dandruff-producing fungus *Malassezia globosa*. Bioorg Med Chem 2017;25:1260–5. 28057408 10.1016/j.bmc.2016.12.040

[23] Nocentini A, Vullo D, Del Prete S, et al. Inhibition of the β-carbonic anhydrase from the dandruff-producing fungus *Malassezia globosa* with monothiocarbamates. J Enzyme Inhib Med Chem 2017;32:1064–70. 28766952 10.1080/14756366.2017.1355307 PMC6010091

[24] Nocentini A, Cadoni R, Dumy P, et al. Carbonic anhydrases from *Trypanosoma cruzi* and *Leishmania donovani chagasi* are inhibited by benzoxaboroles. J Enzyme Inhib Med Chem 2018;33:286–9. 29278948 10.1080/14756366.2017.1414808 PMC6009872

[25] Innocenti A, Hall RA, Schlicker C, et al. Carbonic anhydrase inhibitors. Inhibition of the beta-class enzymes from the fungal pathogens *Candida albicans* and *Cryptococcus neoformans* with aliphatic and aromatic carboxylates. Bioorg Med Chem 2009;17:2654–7. 19297172 10.1016/j.bmc.2009.02.058

[26] Nocentini A, Vullo D, Bartolucci G, et al. N-Nitrosulfonamides: a new chemotype for carbonic anhydrase inhibition. Bioorg Med Chem 2016;24:3612–7. 27290692 10.1016/j.bmc.2016.05.072

[27] Bonardi A, Vermelho AB, da Silva Cardoso V, et al. N-Nitrosulfonamides as carbonic anhydrase inhibitors: a promising chemotype for targeting chagas disease and leishmaniasis. ACS Med Chem Lett 2019;10:413–8. 30996772 10.1021/acsmedchemlett.8b00430 PMC6466549

[28] Nocentini A, Gratteri P, Supuran CT. Phosphorus versus sulfur: discovery of benzenephosphonamidates as versatile sulfonamide-mimic chemotypes acting as carbonic anhydrase inhibitors. Chemistry 2019;25:1188–92. 30411821 10.1002/chem.201805039

[29] Khalifah RG. The carbon dioxide hydration activity of carbonic anhydrase. J Biol Chem 1971;246:2561–73. 4994926

[30] (a) Zhang Z, Lau J, Zhang C, et al. Design, synthesis and evaluation of 18F-labeled cationic carbonic anhydrase IX inhibitors for PET imaging. J Enzyme Inhib Med Chem 2017;32:722–30; 28385087 10.1080/14756366.2017.1308928 PMC6445240

[31] (a) Vermelho AB, da Silva Cardoso V, Ricci Junior E, et al. Nanoemulsions of sulfonamide carbonic anhydrase inhibitors strongly inhibit the growth of *Trypanosoma cruzi*. J Enzyme Inhib Med Chem 2018;33:139–46; 29192555 10.1080/14756366.2017.1405264 PMC7011998

[32] (a) Bua S, Bozdag M, Del Prete S, et al. Mono- and di-thiocarbamate inhibition studies of the δ-carbonic anhydrase TweCAδ from the marine diatom *Thalassiosira weissflogii*. J Enzyme Inhib Med Chem 2018;33:707–13; 29577755 10.1080/14756366.2018.1450400 PMC6010021

[33] (a) Nocentini A, Bonardi A, Gratteri P, et al. Steroids interfere with human carbonic anhydrase activity by using alternative binding mechanisms. J Enzyme Inhib Med Chem 2018;33:1453–9; 30221552 10.1080/14756366.2018.1512597 PMC7011995

[34] Smith W, Audrieth FL. Nitrogen compounds of the phosphoric and phosphonic acids. III. preparation and properties of amides of phenylphosphonic and phenylphosphonothioic acids. J Org Chem 1957;22:265.

[35] (a) Supuran CT. Carbon- versus sulphur-based zinc binding groups for carbonic anhydrase inhibitors? J Enzyme Inhib Med Chem 2018;33:485–95; 29390912 10.1080/14756366.2018.1428572 PMC6009921

